# Supplementary material for: Initiation of B-type starch granules in wheat endosperm requires the plastidial α-glucan phosphorylase PHS1
Source: Plant Cell. 2023 Aug 18;35(11):4091–110. doi: 10.1093/plcell/koad217 (PMC10615211; doi:10.1093/plcell/koad217)
Supplement: koad217_Supplementary_Data [file koad217_supplementary_data.zip › tpc.23.00506Supplemental Figures and Tables.pdf]

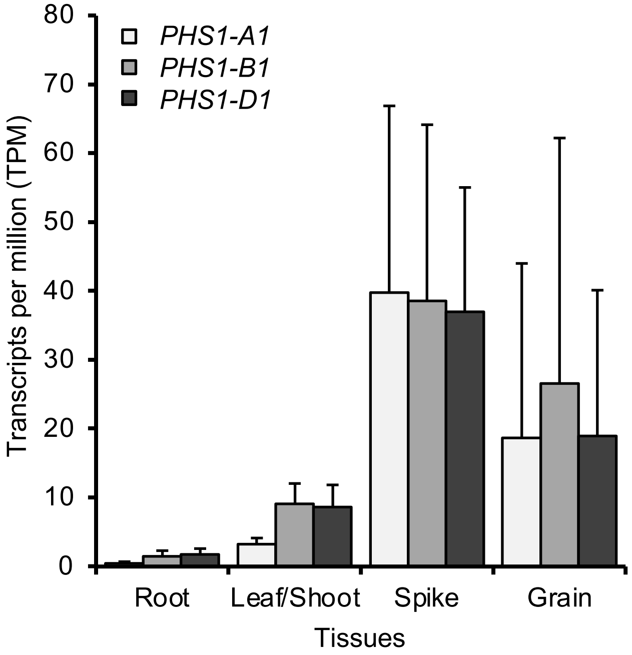

Supplemental Figure S1: Expression levels of PHS1 homoeologs in different tissues of bread wheat. Data are from cultivar Chinese Spring, and obtained using the wheat expression browser (http://www.wheat-expression.com/; Borrill et al. 2016). Error bars represent the SEM. Supports Figure 2.

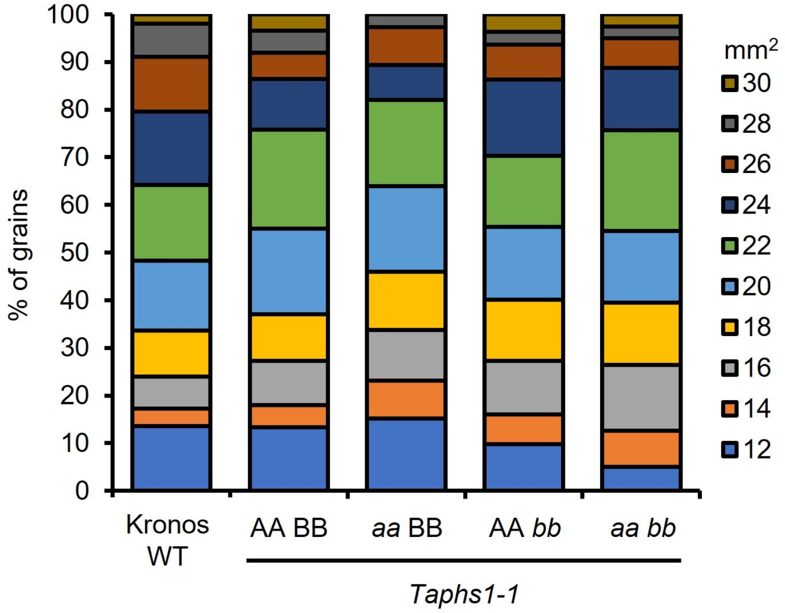

Supplemental Figure S2: Size distribution of grains from *phs1-1* mutants. Grain size data (calculated based on 2-D area) are from the mature grains. Grains from *n* = 5 plants per genotype were pooled for analyzing the size distribution (representing a total of 167–368 individual grains per genotype). Supports Figure 3.

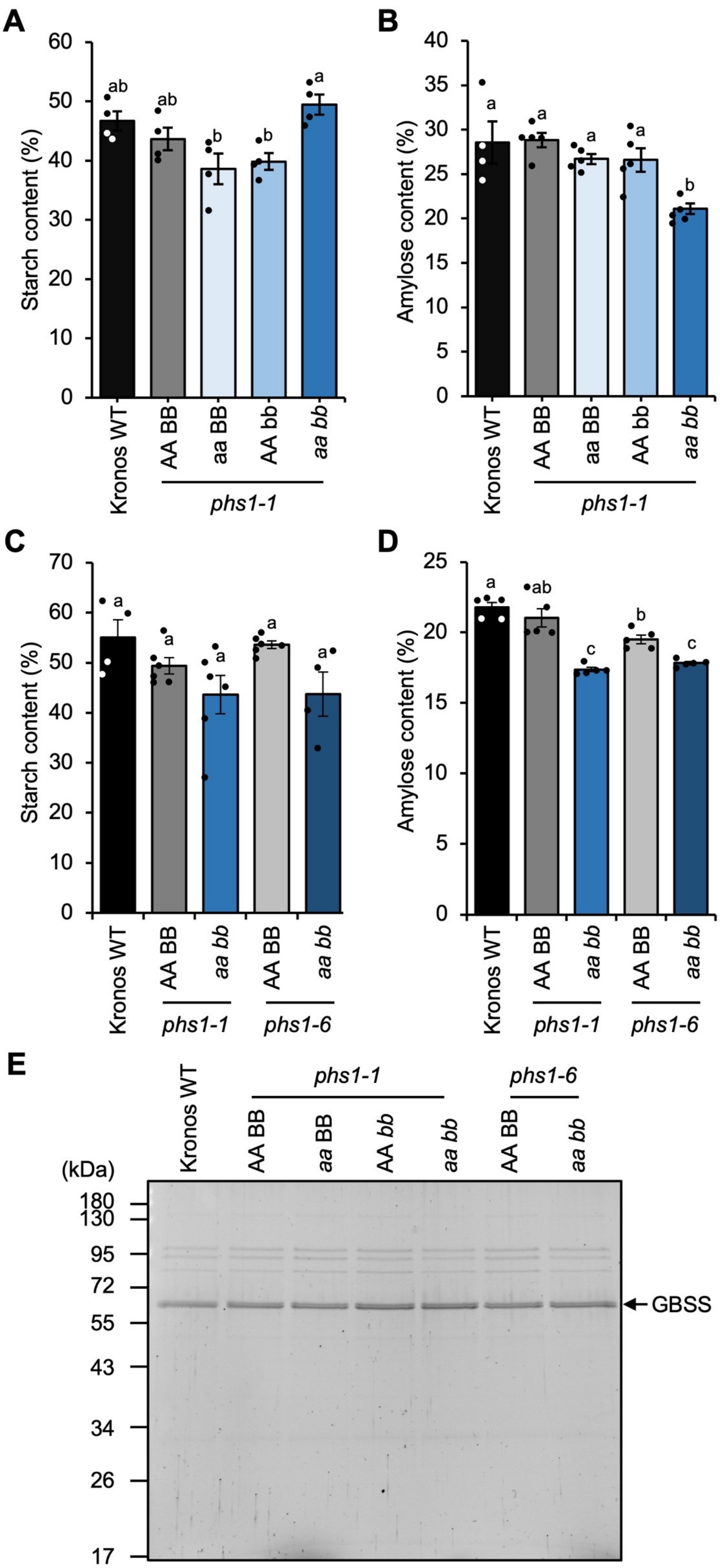
A
B
C
D
E
Starch content (%)
Amylose content (%)
Kronos WT
AA BB
aa BB
AA bb
aa bb
phs1-1
phs1-6
(kDa)
GBSS

Supplemental Figure S3: **Starch and amylose content of *phs1* mutants.** Panels A-D are bar graph representations of the data presented in Table 2. Note that the plants used in Experiment 1 (panels A and B) were grown independently from plants in Experiment 2 (panels C and D). **A** and **C)** Starch content was quantified relative to the dry weight of whole flour. Values are the mean ± SEM on grains from n = 3-6 different plants. **B** and **D)** Amylose content was measured on purified starch. Values are the mean ± SEM on starch extracted from n = 4-5 different plants. Values with different letters are significantly different under a one-way ANOVA and Tukey's posthoc test at $p < 0.05$. **E)** SDS-PAGE of granule-bound proteins stained for total proteins (Sypro Ruby). Lanes were loaded according to equivalent mass of starch (5 mg). The migration of GBSS bands is marked with an arrow. The migration of molecular weight markers (in kiloDaltons, kDa) is indicated on the left of the panel. Supports Table 2.

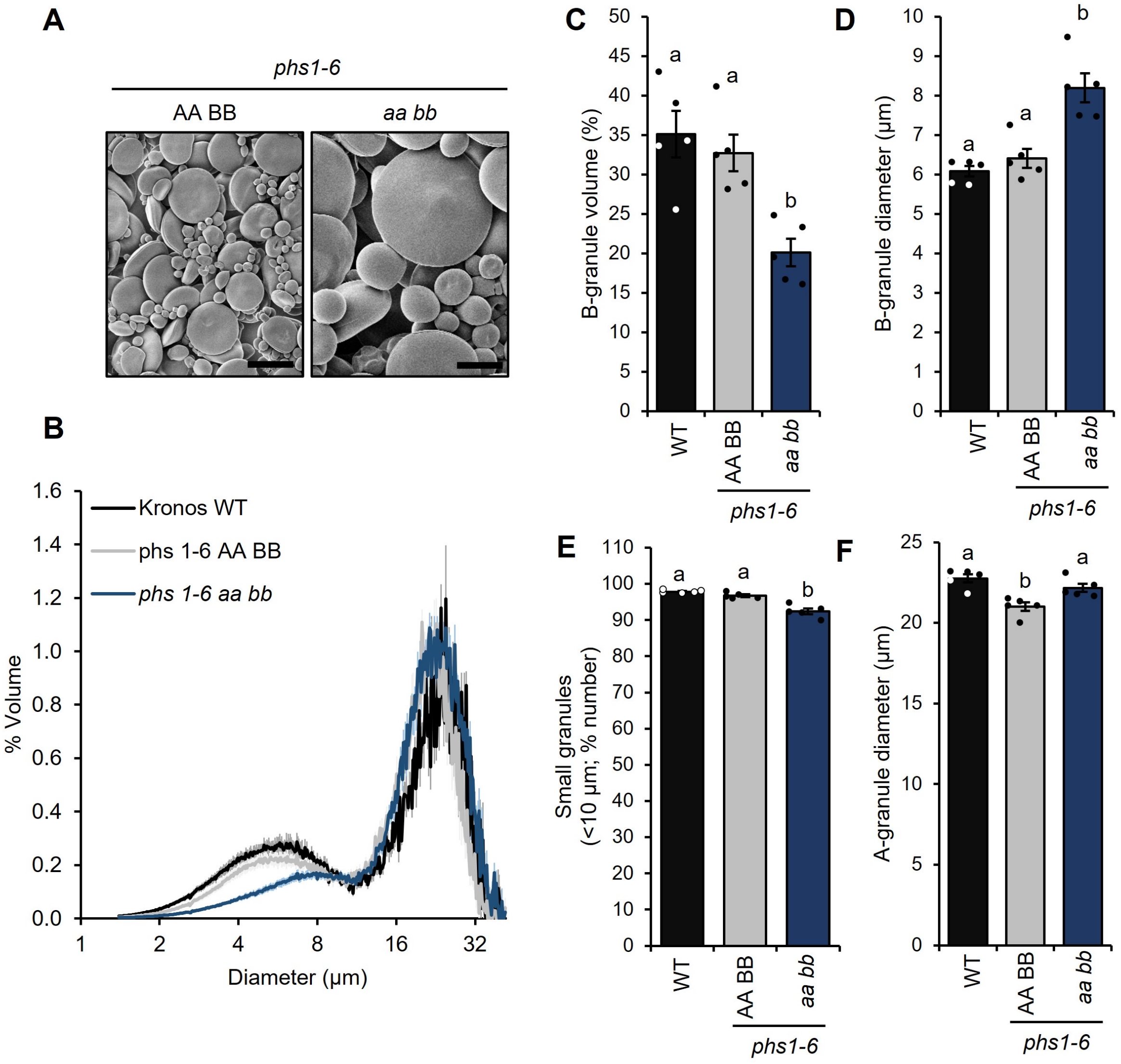

Supplemental Figure S4: **Endosperm starch from the *phs1-6* mutant has fewer and larger B-type granules. A**) Scanning electron micrographs of purified endosperm starch. Bar = 10 µm. **B**) Granule size distributions were determined using a Coulter counter, and the data were expressed as relative % volume (of total starch) vs. granule diameter plots. **C** and **D**) B-type granule volume (% of total starch) and the average diameter of B-type granules were extracted from the relative volume vs. diameter plots by fitting a bimodal mixed normal distribution. **E**) The percentage of small granules by number (smaller than 10 µm) were calculated from the Coulter counter data. **F**) The average diameter of A-type granules was calculated from the relative volume vs. diameter plots, as for panels C and D. For panels B-F, plots show the mean from the analysis of n=5 replicate starch extractions, each from grains from a separate plant. The shading (on panels B) and error bars (on panels C, D, E, F) represent the SEM. Values with different letters are significantly different under a one-way ANOVA with Tukey's post-hoc test ($p < 0.05$). Note that the plants for this experiment were grown independently of the plants used in Figure 4. Supports Figure 4.

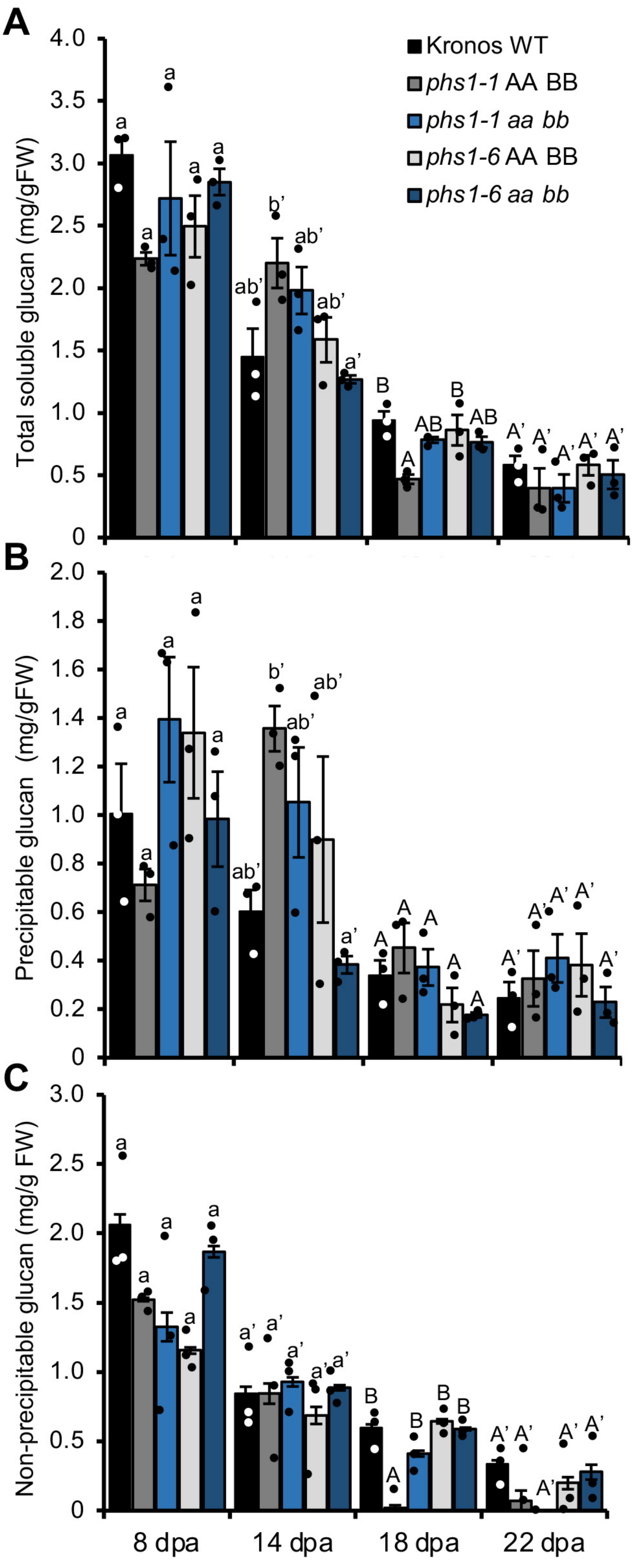
A
Total soluble glucan (mg/gFW)
4.0
3.5
3.0
2.5
2.0
1.5
1.0
0.5
0
Kronos WT
phs1-1 AA BB
phs1-1 aa bb
phs1-6 AA BB
phs1-6 aa bb
B
Precipitable glucan (mg/gFW)
2.0
1.8
1.6
1.4
1.2
1.0
0.8
0.6
0.4
0.2
0
C
Non-precipitable glucan (mg/g FW)
3.0
2.5
2.0
1.5
1.0
0.5
0
8 dpa
14 dpa
18 dpa
22 dpa

Supplemental Figure S5: Soluble glucan quantification in the developing endosperm tissue. A) Total soluble glucans, **B**) methanol precipitable fraction of soluble glucans (phytoglycogen and long MOS), and **C**) the non-precipitable fraction (short MOS) in perchloric acid extracts of grain of WT, *phs1-1* and *phs1-6* double mutants and corresponding wild-type controls, harvested at 8, 14, 18 and 22 days post anthesis (dpa), with n = 3 individual plants for each genotype per time point. Values are expressed relative to the fresh weight of the dissected endosperm. Values with different letters are significantly different under a one-way ANOVA with Tukey's post-hoc test ($p < 0.05$). Supports Figure 5.

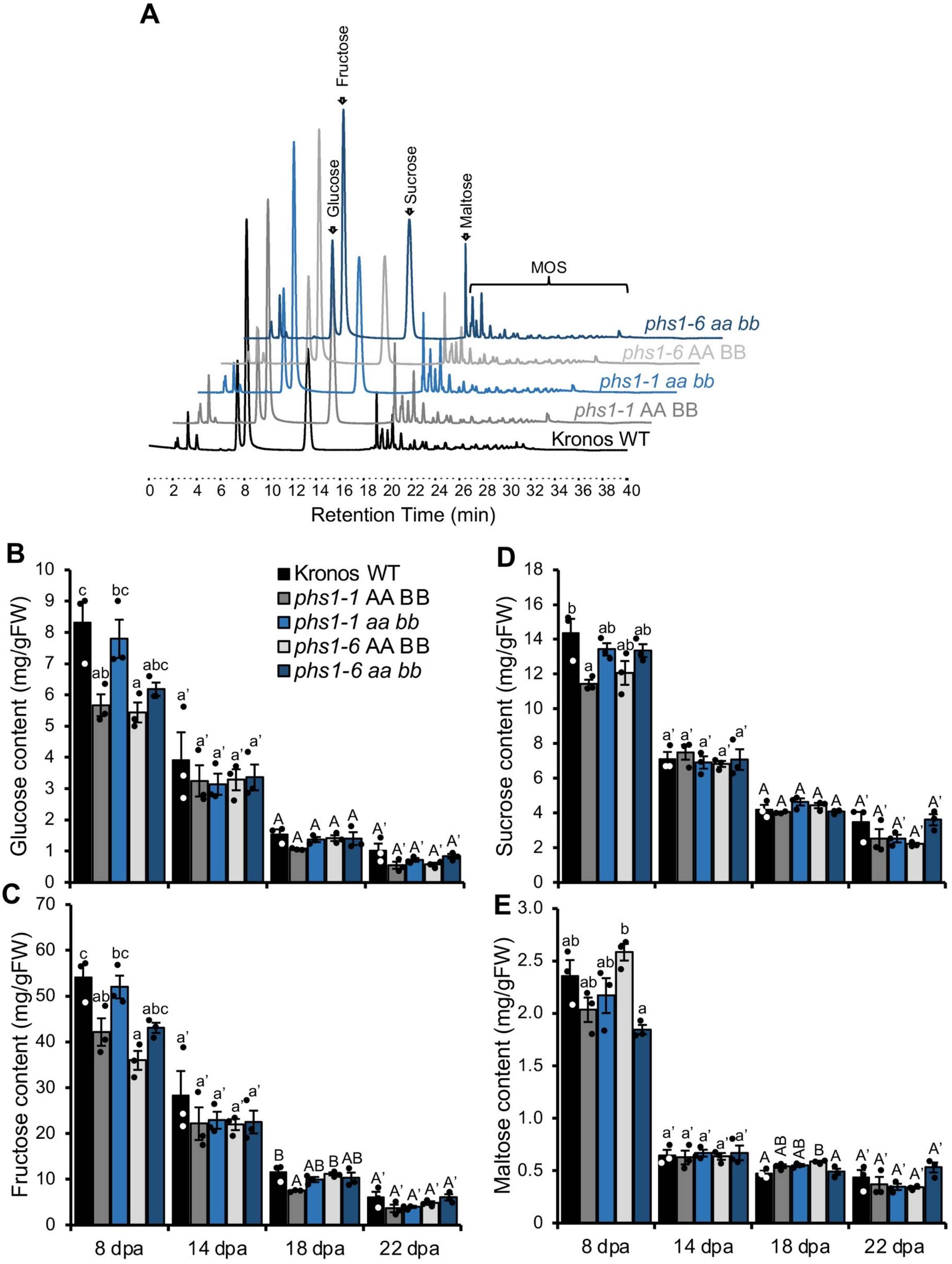

Supplemental Figure S6: **Soluble sugar quantification in the developing endosperm tissue.** **A**) Representative HPAEC-PAD chromatograms, used for the quantification of **B**) glucose, **C**) fructose, **D**) sucrose and **E**) maltose. Perchloric acid extracts of dissected endosperm harvested at 8, 14, 18 and 22 days post anthesis (dpa) were analysed, with n = 3 individual plants for each genotype per time point. Values are expressed relative to the fresh weight of the dissected endosperm. Values with different letters are significantly different under a one-way ANOVA with Tukey's post-hoc test (p < 0.05). Supports Figure 5.

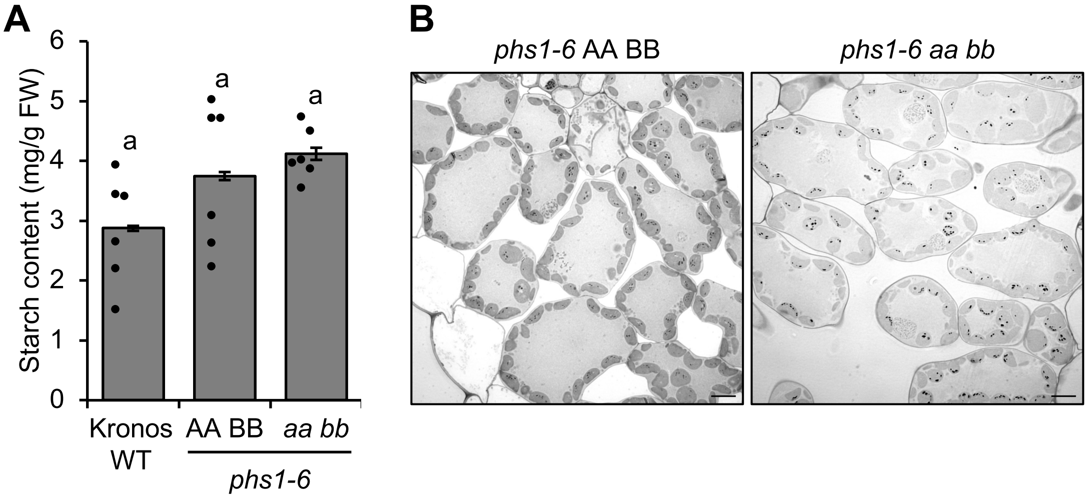

Supplemental Figure S7: Starch granules in leaf chloroplasts of the *phs1-6* mutant. **A)** Total starch content of leaves of the Kronos WT, wild-type segregant control (AA BB) and double mutant (*aa bb*). Seedlings were grown for two weeks under 16 h day/8 h night and harvested at the end of day. Values are the mean ±SEM from n = 6 plants (each represented by a data point). There were no significant differences under a one-way ANOVA and Tukey's posthoc test at $p < 0.05$. **B)** Light micrographs of leaf sections stained with Periodic Acid-Schiff (PAS) staining to visualise starch granules. Leaf segments were harvested halfway along the length of the older blade in two-week-old seedlings were harvested at the ED, prior to fixation and embedding. Bars = 10 µm Supports Figure 8.

Supplemental Table S1: Primers used for KASP genotyping

Allele	WT	mutant	common
K4533	tttttgattctctgatattgaattg	tttttgattctctgatattgaatta	aaggtatgagataaggctgaaga
K4367	atatccgtttcgacctgcag	atatccgtttcgacctgcaa	ccatatgtccttggcatactcg
K2864	agagacatcatttcttacgatctcc	agagacatcatttcttacgatctct	acctgcatgttagcttcttttct
K0238	aaaataaagtagacgaaagaatac	aaaataaagtagacgaaagaatat	acaaaattttgatttgggtgcc
K2244	ttgtcaagagaccatgttcgc	ttgtcaagagaccatgttcgt	ctcctagtcgaagtccaacta
K3239	gcccaatcctgcttcagaag	gcccaatcctgcttcagaaa	gccacccattttgtagttagttag

All primer sequences are provided in 5’ to. 3’ orientation. For KASP genotyping, the VIC/HEX tail (gaaggtcggagtcaacggatt) was added to the 5’ end of the WT allele primers, and the FAM tail (gaaggtgaccaagttcatgct) was added to the 5’ end of the mutant allele primers.

Supplemental Table S2:
Oligonucleotides used in this study.

Cloning of PHS1:pENTR

Forward primer	Reverse primer
CACCATGGCGACCGCCTCGCCGCCGCTCG	GGGCATGATGACGGGGCTGATACC
