# Supplementary material for: Initiation of B-type starch granules in wheat endosperm requires the plastidial α-glucan phosphorylase PHS1
Source: Plant Cell. 2023 Aug 18;35(11):4091–110. doi: 10.1093/plcell/koad217 (PMC10615211; doi:10.1093/plcell/koad217)
Supplement: koad217_Supplementary_Data [file tpc23.00506Peer Review Report.pdf › tpc23.00506Peer Review Report.pdf]

Initiation of B-type starch granules in wheat endosperm requires the plastidial α-glucan phosphorylase PHS1

Nitin Uttam Kamble, Farrukh Makhamadnojov, Brendan Fahy, Carlo Martins, Gerhard Saalbach, David Seung

Corresponding author: Nitin Uttam Kamble (Nitin.Kamble@jic.ac.uk) and David Seung (David.Seung@jic.ac.uk).

Review timeline:

**TPC2023-RA-00506**	Submission received:	June 1, 2023
	1st Decision:	July 14, 2023 *accept with minor revisions*
**TPC2023-RA-00506R1**	1st Revision received:	July 20, 2023
	2nd Decision:	July 28, 2023 *acceptance pending, sent to science editor*
	Final acceptance:	Aug. 8, 2023

REPORT: (The report shows the major requests for revision and author responses. Minor comments for revision and miscellaneous correspondence are not included. The original format may not be reflected in this compilation, but the reviewer comments and author responses are not edited, except to correct minor typographical or spelling errors that could be a source of ambiguity.)

TPC2023-RA-00506 1st Editorial decision – *accept with minor revisions* **July 14, 2023**

We have received reviews of your manuscript entitled "Initiation of B-type starch granules in wheat endosperm requires the plastidial α-glucan phosphorylase PHS1." On the basis of the advice received, the board of reviewing editors would like to accept your manuscript for publication in The Plant Cell. This acceptance is contingent on revision based on the comments of our reviewers. In particular, please consider the following:

The two reviewers just have a number of relatively minor comments and suggestions, most of which can be addressed by appropriate textual changes in your manuscript.

-- Reviewer comments:

[Reviewer comments shown below along with author responses]

TPC2023-RA-00506R1 1st Revision received **July 20, 2023**

Reviewer comments and **author responses**:

Reviewer #1:

PHS1 and PTST2 may interact physically as not very convincingly suggested by affinity pulldown and mass spectrometry. Immunoprecipitation assay carried through heterologous expression of the wheat proteins in Nicotiana plastids are more convincing in this respect. I was not convinced by the absence of SSIV in the pulldown experiments.

> **Response: We agree that the pulldown experiment alone is not sufficient to conclude that PHS1 and BGC1(PTST2) interact, and agree that our assay with the transiently expressed proteins make the conclusion more convincing. Regarding the absence of SS4, we believe the primary reason why we did not identify SS4 in the pulldown is because we used extracts from 18 dpa grains for this analysis. We have shown that SS4 expression is highest at early stages of grain development (i.e., during the initiation of A-type granules), and that its expression decreases during the later stages, particularly during the initiation of B-type granules (Hawkins et al., 2021; Chen et al., 2023). Thus, any interaction between BGC1 and SS4 may be restricted to earlier stages of grain development, when SS4 protein is more highly present (as also raised by Reviewer #2). We have now clarified this in the revised manuscript on Lines 161-165.**

They have selected mutants defective for either the A and B genomes from the durum wheat using in silico TILLING resources. They used either premature nonsense or splice acceptor mutations and combined the two non-sense mutation from the A and B genome to generate the phs1-1 allele and similarly the two-splice acceptor phs1-6 defective alleles for both the A and B genome encoded enzymes. It was not clear to me if the two nonsense mutations were identical or if the splices donor mutations were equally identical. Nevertheless, I found the zymogram analysis of the phosphorylase activity very convincing. However as evident from the above statements, I struggled with the tetraploid genetics, and I would suggest rephrasing their description to make this clearer to the general readership not necessarily familiar with wheat tetraploid genetics.

Response: Thank you very much for this suggestion. We agree that the tetraploid genetics could have been clearer. We have improved the description of the mutants and what the "AA BB" notation represents in the revised manuscript (Lines 217-226).

All mutants had identical starch content in their grains and both types of double mutants did witness a small yet significant decrease in amylose content. The data presented sustain the conclusion. I believe this result would have benefited from additional documentation, notably the measures of GBSS activity and protein in the A or B granules (if possible). I also think that the report or documentation of the unchanged WSP amounts in the grains (MOS + soluble polysaccharides (dextrins)) of the PHS1 mutants is desirable at this stage in the manuscript development (the authors describe this much later prior to the discussion of their data) to understand this decrease in amylose which might also be telling us something about the mode action of phosphorylase. Indeed, MOS are expected to significantly boost amylose synthesis and if PHS1 functions here in the biosynthetic mode then we would expect a negative impact on amylose content. On the other hand, if PHS1 works in degradation then the reverse would be expected (these conclusions hold only if GBSS activity and protein are not significantly modified in the mutants). In any case having an analysis of the soluble glucans in these mutants at this stage would have been very helpful in a paper dealing with the function of PHS1 in B granule seeding. Hence the authors could have concluded here that MOS levels are apparently not the cause of this amylose decrease. Please note that the heading "Loss of PHS1 in wheat does affect total MOS levels in the endosperm" (line 372) is in clear contradiction with the analysis of WSP and MOS reported. I guess this is a typo!

Response: Thank you very much for your positive comments and suggestions. As suggested, we analysed the abundance of GBSS bound to the starch granules in mature grains of *phs1-1* and *phs1-6* double mutants using SDS-PAGE. GBSS levels were not different between the mutants and their corresponding wild-type controls. We have added these data in the new Supplemental Figure 3 and discuss them on Lines 282-294 and Lines 585-598 of the revised manuscript. Analysis of the GBSS specifically in A- and B-type granules was not possible due to the technical challenges in separating the two granule populations. However, we would not expect differences in GBSS abundance between A- and B-type granules since GBSS levels were not altered in the *phs1* mutants, which have a very different ratio of A- and B-type granules compared to the wild-type.

We generally agreed that moving the MOS data to an earlier stage of the manuscript would allow better discussion of MOS and amylose content. Although we tried to make this work, we realised that the original placement of the MOS data was optimal for the narrative, and so we decided not to move the data. The main issue was that the MOS is necessarily measured in developing grains, whereas the starch content and amylose is measured in mature grains. Thus, it made more sense to introduce MOS measurements after the developing grain experiment had been introduced. However, we have extended the discussion in the revised manuscript (Lines 585-598) to better discuss the low amylose content and its links to MOS levels.

Thank you for pointing out our typographical error. We have fixed this to "Loss of PHS1 in wheat does not affect total MOS levels in the endosperm" in the revised manuscript (Line no 424).

Most interestingly the mutants defective for PHS1 show a clear-cut defect in seeding of B-granules, displaying a highly restricted number of much larger B granules. This is the major finding of the paper which I believe is well sustained by the data. I also agree with the authors that this strongly suggests a biosynthetic function of PHS1 replacing that of SSIV in the B-type seeding process (although I am not convinced that SSIV is not around at the time of B-type granule seeding). This is novel information, and such a function has never been demonstrated in other plants or green algae.

Response: Thank you very much for your positive comments. We want to clarify that we have not concluded that SS4 is not around (completely absent) during B-type granule initiation. However, we believe we have provided evidence in previous papers that SS4 levels are lower in later grain development compared to earlier stages, based on expression data and immunoblots (Hawkins et al., 2021; Chen et al., 2023). We hope this is made clearer in lines 161-165 of the revised manuscript.

There are over 40 genes likely involved in the synthesis and degradation of two glucosidic linkages in the green lineage. These genes are well conserved and exert a similar function in starch metabolism in all plants and green algae. The expressivity of mutant phenotypes does change between species but notably also between organs of the same species and dramatically so sometimes. Having several distinct names in different plant species for what is basically the same enzyme is a source of great confusion. I would advise mentioning BGC1 and Flo6 which are the generic phenotype-based names of wheat and rice PTST2 respectively in the introduction only and then switch systematically to the use of PTST2. PTSTs are a biochemically and phylogenetically coherent family of proteins with three distinct well conserved isoforms. Please simplify and only use PTST2.

Response: We fully agree with using more systematic nomenclature across species. However, we also understand and respect the convention of adhering to naming systems that have already been established within species. The name BGC1 for the wheat PTST2 has already been published in Chia et al. (2020) and we therefore believe they have naming priority. Using PTST2 in this manuscript would only generate more confusion. However, it is a very important issue since all starch genes have different names in different species (e.g.: SSIV, SS4; SU2, ISA1, etc). We are enthusiastic about consolidating naming systems in the future, and we will initiate discussions within the starch research community to make a single community-approved naming guideline.

I would like to know what happens to SSIII in this wheat system, as this protein has also been reported to impact starch granule seeding

Response: A previous study by Fahy et al., 2022 in hexaploid wheat suggests that mutation of SSIIIa results in altered size and shape of A-type granules, but no clear effect on B-type granules. We agree with the reviewer that this is an important discussion point, and have now added this information in the revised manuscript (Lines 527-531).

A and B granules are specific to Triticeae. I would like to know what ideas breeders and starch scientists have on the reasons why this specific trait was selected in this group of cereals.

Response: This is an interesting question, for which we unfortunately do not have a clear answer. Understanding the physiological significance of the bimodal granules is one of the major research questions in our group. We believe that the bimodal trait developed early during the divergence of Triticeae: every species we have examined in this tribe (including wild *Agropyron*, *Elymus* and *Aegilops* species) have A- and B-type granules. This strongly suggests that the bimodal trait itself is not a result of human selection, although we may have selected for specific A- and B-type granule ratios within species that already had the trait. Now that we have mutants that lack the B-type granules, we are currently testing subsequent impacts on physiological traits (e.g. grain germination), as well as industrial traits (e.g. grain quality).

<u>**Reviewer #2**</u>:

We are very grateful to the reviewer for reviewing our manuscript, and for their positive response.

1) Regarding the amount of amylose: The phs1-1 aabb line clearly accumulates less amylose than the wild-type lines (phs1-1 AABB et Kronos WT). However, if I understood the statistical analysis correctly, this does not appear to be the case for the phs1-6 aabb line when compared to the wild-type sibling control (phs1-6 AABB). In addition, high differences are observed between the 2 experiments for Kronos WT (28.6% and 21.5%). Can the authors comment on this? Regarding results obtain for phs1-6, the statement that PHS1 is required for normal amylose content should probably be modulated (line 297-298).Also, as written in lines 537-538, it is possible that the lower amylose content is a direct consequence of having low B-Type granules (If we imagine that there is a higher amylose % in B-type granules than in A-type granules). In this case, regarding amylose, can the authors really say that "Loss of PHS1

affects products of MOS extension" (line 500). Finally, since phs1-1 and phs1-6 are different on this point, could the variation in amylose content in phs1-1 be due to background mutations (mentioned by the authors line 221).

> **Response: Thank you very much for your comments on this. We have rechecked the data and statistics on this, and we have realised that we had presented an older version of the analysis for Experiment 2. We have now updated the values of Table 2, but the statistical result remains unchanged. The reviewer is correct that when compared to the *phs1-6* wild-type segregant, the *phs1-6* double mutant is not statistically different, although it is when compared with the other wild-type controls. This is likely because the *phs1-6* wild-type segregant had a slightly lower amylose content when compared to the WT, for unknown reasons. Nevertheless, we have toned down the conclusion about the altered amylose content as suggested, and we now extensively discuss the difference between *phs1-1* and *phs1-6* in Lines 282-294 of the revised manuscript. We have also included a graphical version of Table 2 in Supplemental Figure 3 (as suggested below), where all raw data points can be observed.**
>
> **We agree that the absolute amylose content of WT differs between the two experiments. As noted in the Table 2 legend, the two experiments are using two different batches of plants. We have now also added in the legend that these batches were also grown in two different glass houses. In our experience, absolute values of starch content, amylose content, as well as the B-type granule content tends to vary between experiments – likely due to the different growth environments. However, relative differences between genotypes are consistent.**
>
> **We also fully agree that our paragraph discussing the low amylose content was somewhat contradictory, and that we cannot yet distinguish whether the low amylose content is due to the low B-type granule content or altered MOS metabolism. We therefore made it clear that we have insufficient data to distinguish these possibilities in the revised manuscript (Lines 585-598).**

2) Related to the previous comment on variations in amylose content; the data presented for Kronos WT are very different between Fig 4 and supplemental Figure 3.

Example: B-granule volume (%) is around 23 in fig4 while this value reach 35 in supplemental Fig 3. Are these plants from the same experiment or from different cultures (like in table 2 where 2 distinct experiments are clearly identified)?

> **Response: Thank you so much for pointing this out. Again, we think this is because they are from two different batches of plants grown in different glasshouses. We have now clarified this in the legend of Supplemental Figure 4 in the revised manuscript: "Note that the plants for this experiment were grown independently of the plants used in Figure 4."**

3) Lines 364-365 authors say that they "calculated that the size of A-type granules was unaffected by the phs1 mutations in the bgc1-1 background (figure 7C)."However, when I look at Figure 7C; PHS1xbgc1-1 (in green) seems significantly different from phs1-1xbgc1-1 (dark grey). It is however true that both mutants are not significantly different than the wild-type sibling PHS1xBGC1 (light grey). Can the authors clarify what they are comparing to say that the size of A-type granules is not modified by the phs1 mutations?

> **Response: We agreed with this comment and we have modified the text on Line 410 of the revised manuscript to clearly mention that the comparison is with the wild-type sibling control. In comparison with the wild-type sibling PHS1xBGC1 (which is the most correct control), the single and double mutants have no significant difference in the A-type granule size.**

4) In Figure 2; the authors stated that they identified 4 different phosphorylase activities. One of these activities, unidentified, displayed a reddish color. Can the authors be sure that this band corresponds to a phosphorylase activity? Does this band disappear when the gel is incubated without G1P? Could this activity correspond to another enzyme of starch metabolism (DPE 2 for instance)?

> **Response: Thank you very much for this suggestion. As suggested, we ran the native gel analysis with *phs1-1* and *phs1-6* mutant lines (along with the wild-type siblings) with and without G1P substrate. When gels were incubated without G1P, all bands disappeared, including the unidentified band (see below). This suggests that the band represents PHS activity. Since it cannot be PHS1 activity (it is unaffected by the mutations), we suspect that the band represents another PHS2 activity, perhaps reflecting a different oligomeric state of the enzyme. Further investigation using mass spectrometry will shed more light on this.**

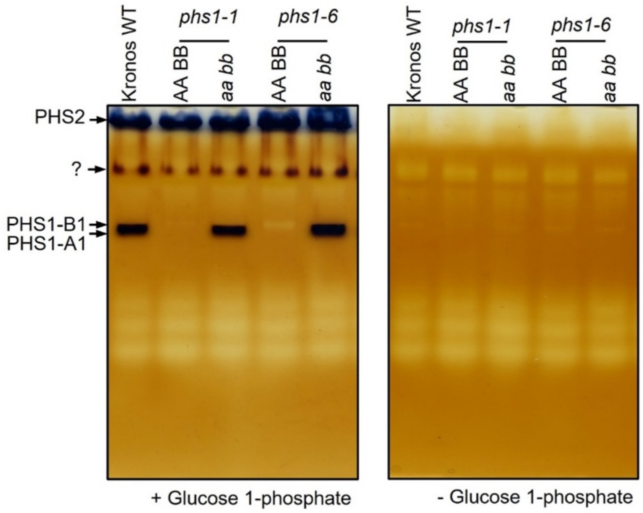

Native PAGE of phosphorylase activity using extracts of leaves from one-month-old plants of the wild type (Kronos WT), *phs1-1* and *phs1-6* double (*aa bb*) mutants, and respective wild-type segregant controls (AA BB). Crude extracts were separated on 7.5% polyacrylamide gels containing 0.3% glycogen (~150 µg protein per well). Activity bands were visualised after incubation with G1P (left panel) and without G1P (right panel) by staining in Lugol's iodine solution.

5) Regarding the data presented in Table 2; Wouldn't it be easier for the reader to represent starch content and amylose content as histograms? This would make it possible to display the different values obtained (as in figure 4c, d,f,g for example). It may also help the reader to visualize why differences in starch content observed in experiment 2 between Kronos WT (55% {plus minus}2) and phs1-1 aabb (44% {plus minus}1) are not statistically significant.

Response: Thank you very much for this suggestion. We have chosen to leave Table 2 in the main manuscript because it presents the data from the two experiments in a compact manner. However, we have also replotted the data graphically in Supplemental Figure 3, including the raw data points, for specialists wishing to do more detailed analyses of the data.

Minor remarks

* it is unfortunate that the data regarding the expression pattern of PHS1 are sometimes established from data generated in durum wheat (expression during endosperm development) and sometimes in bread wheat cultivar (expression in different tissues).

Response: We agreed that ideally, all expression data would come from durum wheat. The data set we generated in the endosperm is from the same tetraploid wheat background as the WT (Chen et al., 2023), but we did not examine leaves in that experiment. However, there is high-quality expression data available for comparing different tissues in hexaploid wheat on the wheat expression browser, but this does not include a detailed developmental timecourse of gene expression in the starchy endosperm. This is why both datasets were used in our analyses.

* Please clarify what the error bars in Supplementary Figure 1 correspond to (SE, SD?)

Response: Thank you for the pointing out the missing information. Error bars in Supplementary Figure 1 represent the SEM. We have now added this detail to the legend.

* In the pulldown experiment using His-tagged BGC1, many proteins interacting with BGC1 were identified by mass spectrometry. However, although SS4 has previously been described as interacting with BGC1, no peptide corresponding to SS4 was detected during this experiment.do the authors have any assumptions about this absence? Is it possible that at 18 DPA SS4 is only expressed at a very low level, making it difficult to identify by pulldown? Is the interaction between SS4 and BGC1 dependent on a particular developmental stage or organ?

Response: Thank you very much. As stated in our response to Reviewer #1 above, we absolutely agree that the

timepoint investigated could be one of the main reasons why we did not find SS4. We have now clarified this in the revised manuscript on Lines 161-165.

TPC2023-RA-00506R1 2nd Editorial decision – *acceptance pending* **July 28, 2023**

We are pleased to inform you that your paper entitled "Initiation of B-type starch granules in wheat endosperm requires the plastidial α-glucan phosphorylase PHS1" has been accepted for publication in The Plant Cell, pending a final editorial review by a science editor.

Final acceptance from Science Editor **Aug. 8, 2023**
